# Are interventions to increase the uptake of screening for cardiovascular disease risk factors effective? A systematic review and meta-analysis

**DOI:** 10.1186/s12875-016-0579-8

**Published:** 2017-01-17

**Authors:** AT Cheong, SM Liew, EM Khoo, NF Mohd Zaidi, K Chinna

**Affiliations:** 1 Department of Primary Care Medicine, University of Malaya Primary Care Research Group (UMPCRG), Faculty of Medicine, University of Malaya, 50603 Kuala Lumpur, Malaysia; 2 Department of Family Medicine, Faculty of Medicine and Health Sciences, Universiti Putra Malaysia, 43400 Serdang, Selangor Malaysia; 3 Department of Social and Preventive Medicine, Faculty of Medicine, University of Malaya, 50603 Kuala Lumpur, Malaysia

**Keywords:** Systematic review, Meta-analysis, Intervention, Cardiovascular, Screening, Prevention

## Abstract

**Background:**

Cardiovascular disease (CVD) is the leading cause of death globally. However, many individuals are unaware of their CVD risk factors. The objective of this systematic review is to determine the effectiveness of existing intervention strategies to increase uptake of CVD risk factors screening.

**Methods:**

A systematic search was conducted through Pubmed, CINAHL, EMBASE and Cochrane Central Register of Controlled Trials. Additional articles were located through cross-checking of the references list and bibliography citations of the included studies and previous review papers. We included intervention studies with controlled or baseline comparison groups that were conducted in primary care practices or the community, targeted at adult populations (randomized controlled trials, non-randomized trials with controlled groups and pre- and post-intervention studies). The interventions were targeted either at individuals, communities, health care professionals or the health-care system. The main outcome of interest was the relative risk (RR) of screening uptake rates due to the intervention.

**Results:**

We included 21 studies in the meta-analysis. The risk of bias for randomization was low to medium in the randomized controlled trials, except for one, and high in the non-randomized trials. Two analyses were performed; optimistic (using the highest effect sizes) and pessimistic (using the lowest effect sizes). Overall, interventions were shown to increase the uptake of screening for CVD risk factors (RR 1.443; 95% CI 1.264 to 1.648 for pessimistic analysis and RR 1.680; 95% CI 1.420 to 1.988 for optimistic analysis). Effective interventions that increased screening participation included: use of physician reminders (RR ranged between 1.392; 95% CI 1.192 to 1.625, and 1.471; 95% CI 1.304 to 1.660), use of dedicated personnel (RR ranged between 1.510; 95% CI 1.014 to 2.247, and 2.536; 95% CI 1.297 to 4.960) and provision of financial incentives for screening (RR 1.462; 95% CI 1.068 to 2.000). Meta-regression analysis showed that the effect of CVD risk factors screening uptake was not associated with study design, types of population nor types of interventions.

**Conclusions:**

Interventions using physician reminders, using dedicated personnel to deliver screening, and provision of financial incentives were found to be effective in increasing CVD risk factors screening uptake.

**Electronic supplementary material:**

The online version of this article (doi:10.1186/s12875-016-0579-8) contains supplementary material, which is available to authorized users.

## Background

A major challenge to the control of cardiovascular disease (CVD) is the high prevalence of cardiovascular risk factors such as hypertension, diabetes and obesity [1–3]. A substantial proportion of the global population remain unaware of their existing cardiovascular risk factors [4–6]. Modifiable risk factors for CVD account for 90% of the risk of myocardial infarction, which indicates that CVD is largely preventable [7]. Effective management strategies such as lifestyle changes or pharmacotherapy are available to modify these risk factors, which have been shown to reduce CVD morbidity and mortality especially for those at high risk [8–12]. Two cohort studies from Korea and Japan reported that health screening for CVD was associated with lower rates of CVD, all-cause mortality, CVD events and lower healthcare utilization and costs [13, 14].

There has been considerable debate regarding the usefulness of screening for CVD risk factors [15–19]. A systematic review by Krogsbøll found that general health checks did not reduce morbidity or mortality of CVD [20]. Others have argued that the results of this review cannot be generalized because it included old studies from an era when management was not as effective as current treatment [21]. As the review also focused on general health checks, the findings may differ from health checks conducted for specific conditions such as CVD and cancer [21–23].

Various strategies and interventions have been used to increase individuals’ participation in CVD risk factors screening. Their effectiveness varied from study to study, ranging from no benefit to an 80% increase in the participation rate from baseline [24–27]. Jepson et al. conducted a comprehensive systematic review to examine factors associated with the uptake of screening programmes and to assess the effectiveness of methods used to increase uptake [28]. However, the majority of the studies included in this review were related to cancer screening, with very limited studies on CVD risk factors screening. To the best of our knowledge, there has previously been no systematic review analyzing the effectiveness of interventions used to increase uptake rate of CVD risk factors screening amongst the general population from primary care practices and the community. Previous systematic reviews have focused on assessing the effectiveness of using community pharmacies as the site for CVD risk factors screening [29], the evaluation of behavioural components used in the intervention of screening programmes [30], and reviewing screening approaches in primary care [31]. Therefore, the objective of this study is to examine the literature to determine the effectiveness of interventions used to increase the uptake of CVD risk factors screening in adult population from primary care practices and the community.

## Methods

The research question addressed in this systematic review is as follows:

What is the effectiveness of interventions that aim to increase the uptake of CVD risk factors screening?

- Population: Adults aged 18 years and above
- Intervention: Interventions that aim to increase participation of individuals to screen for CVD risk factors
- Comparison: comparator groups with usual care
- Outcome: uptake rate (participation rate by public or patients or screening rate which was represented by the tests performed by physicians)

### Criteria for study selection

#### Types of studies

Studies on interventions that aimed to increase participation of individuals to screen for CVD risk factors were included. Study designs included randomized controlled trials (RCTs), quasi-RCTs, non-randomized trials with controlled group and studies which used baseline data as the control group (pre- and post-studies). Studies comparing different interventions were excluded if there was no controlled or baseline group.

#### Assessment for screening of CVD risk factors

The CVD risk factors screening included for assessment in the review were measurements of blood pressure (BP), weight, body mass index (BMI), waist circumference (WC), glucose, lipids, total cardiovascular risk score and history taking regarding smoking, physical activity, or nutritional intake. These CVD risk factors screening could have been carried out in a program specifically targeting CVD risk factors screening, or as part of a program with other preventive services such as cancer screening and vaccination.

#### Study population

The population for included studies involved those individuals aged 18 years and above recruited from attendees of primary care practices or the community. We included studies with mixed populations with or without known CVD and studies limited to populations without known CVD.

Studies which targeted specific populations or conditions such as safety screening for sports or exercise participation, gestational diabetes or post partum screening, familial hypercholesterolaemia and participants with a defined condition such as mental disabilities or rheumatoid arthritis, were excluded.

#### Types of interventions

All types of interventions or strategies to increase participation of CVD risk factors screening were included, regardless of whether they were targeted at individual, community, health-care provider or health-care system level.

#### Types of outcome measures

The CVD risk factors screening uptake was measured by 1) participants’ attendance rate for screening, or 2) screening rate by health-care providers. We excluded studies that only reported on the intention to participate or physicians’ compliance with the prescription. In cases where the studies had included screening for health conditions other than CVD risk factors, we chose to report only outcomes related to CVD risk factors.

#### Search methods

A systematic search was conducted using four electronic databases: PubMed (12 June 2014), CINAHL (3 July 2014), EMBASE (10 July 2014), and Cochrane Central Register of Controlled Trials (3 July 2014). Updates of this search strategy were obtained from PubMed weekly until August 2015 and no new study was identified that fitted our inclusion criteria.

A mixture of medical subject headings (MeSH terms) and free text was used for the concept of “cardiovascular”, “uptake” and “screening”. The search strategy for PubMed is shown in Table 1. These search terms and limits were modified accordingly for each individual database, in order to meet its specification. Limits applied were English language and adult population. There was no limit applied for the year of publication. Additional articles were located through cross-checking of reference lists and bibliography citations of the included studies. This reference list and bibliography citations included review papers. For these review papers, we checked the reference lists to retrieve relevant studies.

**Table 1:** Search strategy in PubMed

#1 Search (((((((("Hyperlipidemias"[Mesh]) OR "Cardiovascular Diseases"[Mesh]) OR "Hypertension"[Mesh]) OR "Diabetes Mellitus"[Mesh])) OR ((((((((((cardiovascular[Text Word]) OR coronary[Text Word]) OR stroke[Text Word]) OR heart[Text Word]) OR family history[Text Word]) OR early cardiovascular death[Text Word]) OR hyperlipidemias[Text Word]) OR diabetes mellitus) OR hypertension))) #2 Search ((((((((general practice[Text Word]) OR preventive health service[Text Word]) OR health check*[Text Word]) OR mass screening[Text Word]) OR opportunistic screening[Text Word])) OR (((((health check*) OR "General Practice"[Mesh]) OR "Preventive Health Services"[Mesh]) OR "Mass Screening"[Mesh])))) OR screening[Text Word] #3 Search ((((((((("Patient Acceptance of Health Care"[Mesh]) OR "Patient Participation"[Mesh]) OR "Consumer Participation"[Mesh]) OR "Refusal to Participate"[Mesh]) OR uptak*))) OR (((((patient participation[Text Word]) OR consumer participation[Text Word]) OR uptak*[Text Word]) OR patient acceptance of health care[Text Word]) OR refusal to participate[Text Word]))) OR participat* #4 Search #1AND #2 AND #3	

### Data collection and analysis

#### Study selection and data extraction

Two reviewers (ATC and NFMZ) screened the titles and abstracts of the articles and conference proceedings. Full papers were then retrieved for potentially eligible articles and reviewed for relevance by ATC and NFMZ. An article was included when there was agreement on the fulfilment of the inclusion criteria. In circumstances where there was a discrepancy, discussions with other team members (SML and EMK) were held to reach a consensus. When further details of the paper were required, corresponding authors were contacted via email; for example when full details of the numerator and denominator of the screening rate were not available, or when there was uncertainty about the same data being presented in more than one publication that described a single study, to avoid problems of double counting of subjects in the meta-analysis [27, 32–40].

Data were then extracted independently by two reviewers (ATC and NFMZ) from the included studies. Relevant information extracted included author(s), year, country of study, title, type of setting, type of screening assessment, study design, study population characteristics, type of intervention and the proportion of participation (numerators and denominators) in the intervention and the controlled arm, which represented the CVD risk factors screening uptake rate. Information on study design, study population characteristics, and types of intervention were extracted to allow for meta-regression.

#### Assessment of quality

Appraisal of the quality of methodology of the included studies was conducted using The Cochrane Collaboration’s “Risk of bias” tool [41]. Each study was assessed based on the features of selection bias, performance bias, detection bias, attrition bias, reporting bias and other potential sources of bias for quality of methodology [41]. The quality of descriptions of interventions in publications was assessed using the Template for Intervention Description and Replication (TIDieR) checklist and guide [42]. The TIDier checklist has 12 items to assess the reproducibility of the intervention based on the description, which included items such as brief name, why, what (materials), what (procedure), who provided, how, where, when and how much, tailoring, modifications, how well (planned), how well (actual) [42].

### Data synthesis and analysis

In this review, two analyses were conducted. First, an analysis was carried out on the overall effectiveness of screening uptake of the interventions compared with its control. Second, subgroup analysis was done measuring the effectiveness of screening uptake by study design and type of intervention. Relative risk (RR) with 95% confidence interval (95% CI) was performed for all sets of comparisons. Data from relevant studies were pooled using a random-effects model with OpenMetaAnalyst software [43, 44]. A test of heterogeneity (Cochran’s Q statistic) with reported p-value was performed and the degree of inconsistency across studies was quantified using *I* ^*2*^ [45].

Meta-regression is a method used to explore heterogeneity seen in meta-analysis by examining differences between studies by effect modifiers [46]. In this study, meta-regression was performed in order to explore whether the differences in study designs (RCT, Controlled trial, pre- and post-studies), types of population (no known CVD, mixed population of known and unknown CVD) and types of intervention (physician reminder, patient invitation, using financial incentives, using dedicated personnel and multifaceted approach) could explain the heterogeneity. Random-effects meta-regression was performed using OpenMetaAnalyst software [43].

Some studies compared more than one type of intervention with usual care [27, 47–49]. Each of these intervention groups was analyzed independently and compared with the group with usual care. For studies with separate screening uptake rates for the different risk factors, the outcome could be represented by any one of these rates [27, 33, 50–57]. For example, the study by Harari et al. reported the uptake rate for BP, cholesterol and blood glucose separately [52]. In order to provide a range of the effectiveness of such interventions, two meta-analyses were performed; one pooling the highest effect sizes of the uptake rate (hereon referred to as optimistic) and the other pooling the lowest effect sizes of the uptake rate (hereon referred to as pessimistic). In studies that reported results of screening uptakes using different time periods, we used the longest duration of timeline in the analysis [48, 56].

## Results

### Literature retrieval process

**Fig. 1:**
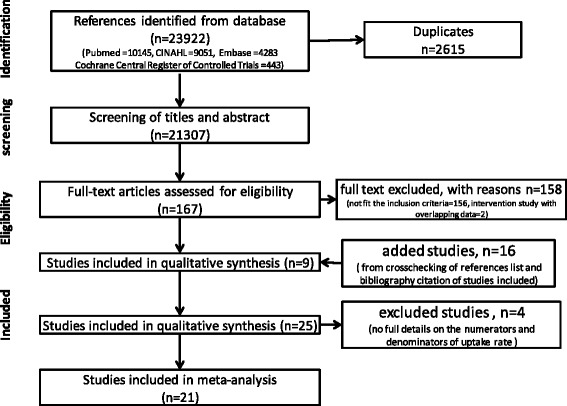
Flow chart of the study search and selection

The search strategy identified 21,307 citations from four databases after removing duplicates. After screening the titles and abstracts, 167 full papers were retrieved for assessment for eligibility. Of these, 158 papers were excluded as they did not fulfill the inclusion criteria. The reasons for exclusion included the age of the study population, the absence of a controlled group, or the outcomes were not related to screening uptake. One study published three papers from data obtained at different periods [32–34], and the most recent paper was included [33]. A total of 9 studies fulfilled the inclusion criteria and were included [27, 33, 35, 36, 47, 54, 55, 58, 59]. Forward and backward searches of the reference lists and bibliography citations of the 9 studies yielded an additional 16 studies [37, 39, 48–53, 56, 57, 60–65] and resulted in a total of 25 studies for qualitative synthesis. We contacted authors of six studies which did not have full details of the numerators and denominators of the screening uptake rates required for meta-analysis. Two authors provided the requested information [27, 37] while authors of the other four studies were either not contactable or stated that they no longer had access to the data. These four studies [35, 36, 39, 65] were excluded, and the final number of studies included in the meta-analysis was 21. Figure 1 illustrates the process of study search and selection.

### Study characteristics of the studies included

Among the 21 studies, ten were randomized or cluster randomized controlled trials [27, 37, 47–52, 58, 60], six were non-randomized trials with controlled group [53–55, 59, 61, 62] and five were pre and post- studies [33, 56, 57, 63, 64]. Eleven studies analyzed CVD risk factors screening [33, 37, 47, 49, 53, 58–63] and 10 studies focused on multiple preventive services including screening for cancer, vitamin B_12_, vaccination promotion, and others [27, 48, 50–52, 54–57, 64] (refer to Table 2). The follow-up period of these studies ranged from 2 months to 3 years. Out of the 21 studies, eight were conducted in Europe (five in the United Kingdom [50, 52, 53, 60, 61], one each in the Netherlands [49], Denmark [59] and Sweden [54]), seven in North America (five in the United States of America [48, 51, 57, 62, 64], two in Canada [27, 47]), three in Australia [55, 56, 58], two in New Zealand [37, 63] and one in Singapore [33].

**Table 2:** Overview of the studies included in the review

Study (Author, year, country)	Population	Type of screening	Setting	Intervention	Related CVR Outcome measures for uptake rate
Randomized/cluster randomized controlled trials					
McDowell, 1989 Canada [47]	Adults aged ≥18 years^a^	BP	Primary care practice	3 intervention groups: 1. Physician reminder (computer-based reminder) 2. Patient reminder (telephone) 3. Patient reminder (letter) Control: usual care	BP
Robson, 1989 UK [50]	Patients aged 30–64 years^a^	Multiple screening	Primary care practice	Dedicated personnel: health promotion nurse Control: doctor worked alone	BP, smoking history, cholesterol, family history of heart attack
Ornstein, 1991 USA [48]	Patients aged ≥18 years^a^	Multiple screening	Primary care practice	3 intervention groups: 1. Physician reminder (paper based) 2. patient reminder (letter) 3. physician and patient reminder (multifaceted approach) Control: usual care	Cholesterol
Apkon, 2005 USA [51]	Patients aged ≥18 years^a^	Multiple screening	Primary care practice	Physician reminders (computer based) vs. usual care	Lipid, smoking screening
Kenealy, 2005 New Zealand [37]	Adults aged 50 years or older^a^	Diabetes screening	Primary care practice	3 intervention arms: 1. Physician reminder (flash alert in computer screen) 2. Physician reminder (patient giving the completed diabetes risk self-assessment form to the doctor) 3. Both 1 & 2 (multifaceted approach) Control: usual care	Glucose^b^
Harari, 2008 UK [52]	Adults aged ≥65 years^a^	Multiple screening	Primary care practice	Health Risk Appraisal via mailed questionnaire and feedback to participants and general practitioners (multifaceted approach) vs. usual care	BP, cholesterol, blood glucose
van Wyk, 2008 Netherland [49]	Men aged 18 to 70 years and women aged 18 to 75 years^a^	cholesterol	Primary care practice	2 intervention for physician reminder (computer based) vs. usual care: 1. Auto-alert 2. On demand alert	Cholesterol
Holt, 2010 UK [60]	patients aged 50–74 years identified as probable high-risk	CVRs	Primary care practice	Physicians reminder (computer based screen alerts vs. usual care	Overall CVRs
Stocks, 2012 Australia [58]	Patients aged 40–74 years	CVRs	Primary care practice	Financial incentives (added voucher incentives) vs. usual care (free test)	Overall CVRs
Grunfeld, 2013 Canada [27]	Adults aged 40–65 years	Multiple screening	Primary care practice	3 intervention arms: 1. Dedicated personnel (practice facilitator at practice level) 2. Dedicated personnel (prevention practitioner at patient level) 3. Multifaceted approach (both 1&2) control: usual care	FBS, BP, Framingham risk calculated, BMI, waist circumference, smoking, physical activity, nutrition^b^
Non-randomized trials with controlled group					
Fullard, 1987 UK [53]	Patients aged 35–64 years^a^	CVRs	Primary care practice	Multifaceted approach (practice facilitator with a practice prevention nurse) vs. usual care	Weight, BP, and smoking history
Franks, 1991 USA [62]	Patients aged ≥18 years^a^	cholesterol	Primary care practice	Financial incentives: Free vs. usual care (paid)	Cholesterol
Christensen, 1995 Denmark [59]	Men aged 40-49	CVRs	Primary care practice	Financial incentives: Free vs. paid	Overall CVRS
Toth-Pal, 2004 Sweden [54]	Adults aged ≥70 years	Multiple screening	Primary care practice	Physician reminder (computer-based) vs. usual care	BP, diabetes
Frank, 2004 Australia [55]	Eligible adults fulfilled screening^a^	Multiple screening	Primary care practice	Physician reminder (computer-based) vs. usual care	Weight, smoking status, BP, diabetes, lipid
Marshall, 2008 UK [61]	patients aged 35–74 years identified as probable high-risk	CVRs	Primary care practice	Dedicated personnel (project nurse) vs. usual care	Overall CVRs
Pre- and Post- studies					
Vincent, 1995 USA [64]	Adult population^a^	Multiple screening	Primary care practice	multifaceted approach: computer generated worksheet with a reminder on health maintenance procedure, periodic physician performance report, patients reminder (letter invitation)	Cholesterol
Bailie, 2003 Australia [56]	≥50 years (majority indigenous)	Multiple screening	Primary care practice	multifaceted approach: clinical guidelines, computerised reminder systems, audit and feedback	Weight, BP, waistcircumference, BMI, glucose
Sinclair, 2006 New Zealand [63]	Adults eligible for cardiovascular risk screening^a^	CVRs	Primary care practice	Multifaceted approach: 1) practice management software enhancement (alert to identify eligible patients for screening, electronic cardiovascular risk assessment tool) 2) CME for the clinical champion who oversee the project 3) Provision of relevant reporting and feedback 4) Eligible patients: letter invitation	Completed cardiovascular risk screen (5-year absolute cardiovascular risk)
wee, 2013 Singapore [33]	Adults aged ≥40 years	CVRs	community	Multifaceted approach: free screening and convenient screening at housing estate	BP, fasting blood glucose and lipid
Butala, 2013 USA [57]	adults^a^	Multiple screening	Primary care practice	Physician reminders (paper-based notes for recommended preventive services)	Lipid and glucose

*BP* blood pressure, *CVRS* uptake for cardiovascular risk factors as a whole

^a^mixed population which consisted of those with known and unknown CVD

^b^results provided by the corresponding author

The interventions for CVD risk factors screening were classified into five types based on their characteristics, adapted from the classification by Jepson et al. [28] whenever possible. These were: (1) physician reminder (paper-based and computer-based reminder), (2) patient invitation (letter and telephone invitation), (3) financial incentives, (4) using dedicated personnel such as project nurse and practice facilitator to help organize and/or carry out screening, and (5) using a multifaceted approach targeting both physicians and patients for screening or using more than one measure to target a population for screening. Physician reminder, patient invitation and financial incentives for screening were interventions that could influence provider or patient behaviour directly or indirectly while using dedicated personnel targeted at provider and organizational level. Multifaceted approaches were targeted at either behavioural or organizational level or both. Table 2 represents an overview of the characteristics of the included studies. Details of the interventions, screening uptake rates (for different risk factors and periods) and the types of assessment are provided in Additional file 1.

### Quality assessment of the studies included

#### Quality of methodology

**Fig. 2:**
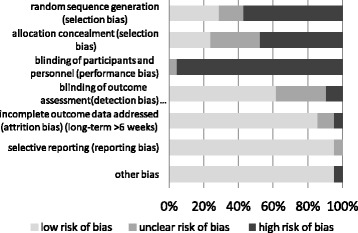
Proportion of studies with low, unclear and high risk of bias

The risk of bias for randomization was unclear and low in 9 out of 10 of the randomized controlled trials, except for one which was allocated high risk as the randomization used odd or even numbers of the last digit of the registration number – a process that was not true randomization [60]. The risk of bias for randomization was high for all non-randomized trials. A description of allocation concealment was presented in five (24%) studies [37, 49, 52, 59, 60]. Blinding of participants and personnel was lacking in all studies due to the nature of the interventions, which involved the participants or health-care professionals directly. The risk of bias for blinding of outcome assessment was low in 13 (62%) studies as the outcome measured in most studies was generated from electronic record systems. One study was found to have a high risk of bias for risk of incomplete outcome data: data from two practices could not be extracted and analyzed [49]. For reporting bias, low risk of bias was found in all studies except the study by Frank et al. where it was unclear [55]. For other biases, the validity of one of the studies needed to be interpreted with caution as one of the authors was related to the company that programmed the trial software [60]. The proportion of studies with low, unclear and high risk of bias is presented in Fig. 2. The summary of risk of bias for individual studies is provided in Additional file 2.

#### Quality of intervention: description and replication

Most studies were clear in the descriptions of the interventions. Two studies lacked descriptions of the education and training materials [33, 53]. Another two studies were unclear on whether training and feedback were provided to the groups [27, 56]. Details of the descriptions of the interventions are provided in Additional file 3.

### Overall effect of interventions compared with controls

**Fig. 3:**
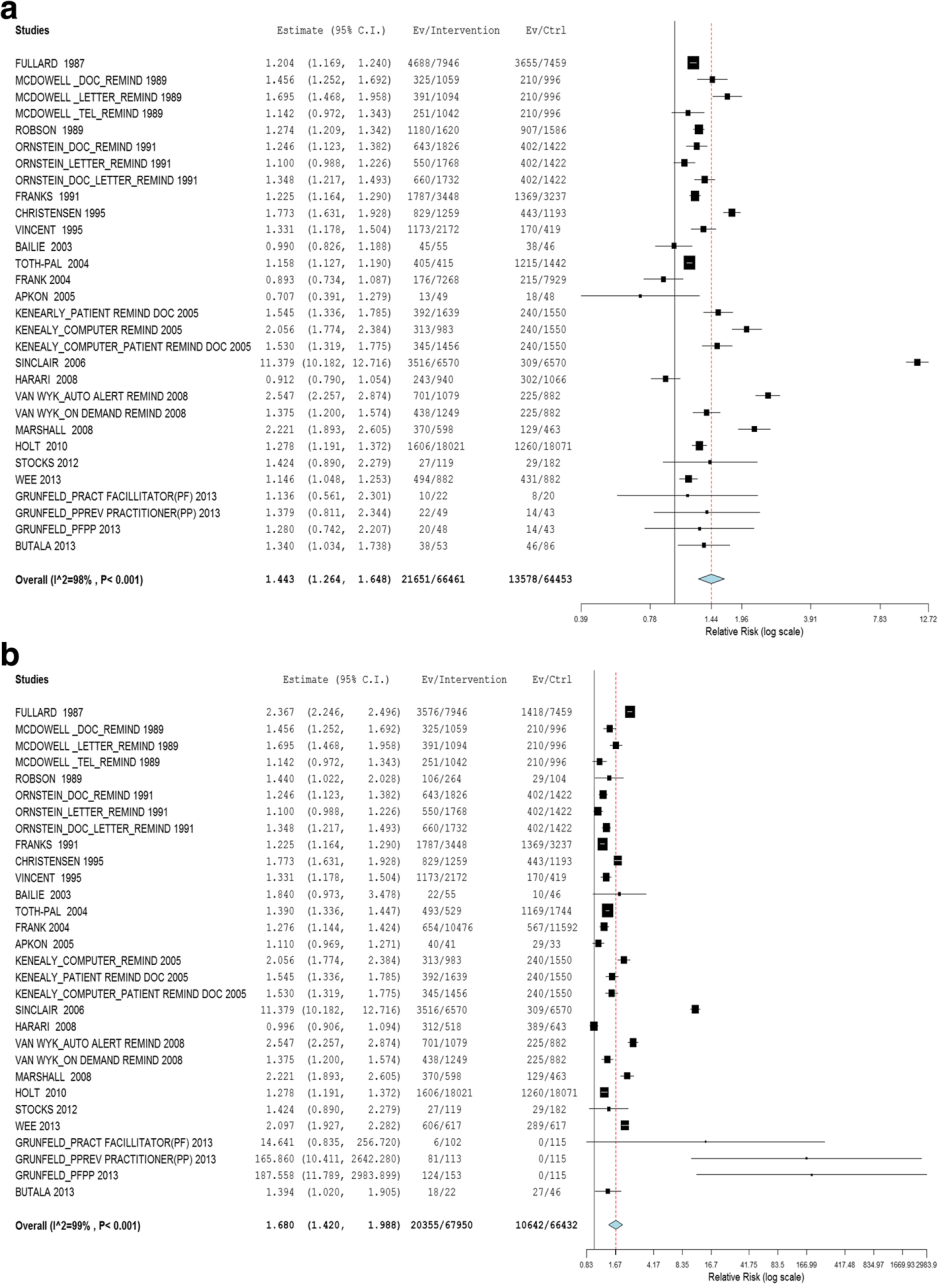
**a** Effect of interventions vs. controls (using lowest effect size as outcome measure). **b** Effect of interventions vs. controls (using highest effect size as outcome measure)

Based on the pooled estimate of the effects of interventions, in both optimistic and pessimistic analyses, the CVD risk factors screening uptake rate was higher in the intervention groups compared with the controls. The RR was 1.443 (95% CI 1.264 to 1.648) using the pessimistic estimate and 1.680 (95% CI 1.420 to 1.988) using the optimistic estimate (refer to Fig. 3a & b).

### Subgroup analyses

#### Effects of study designs

**Fig. 4:**
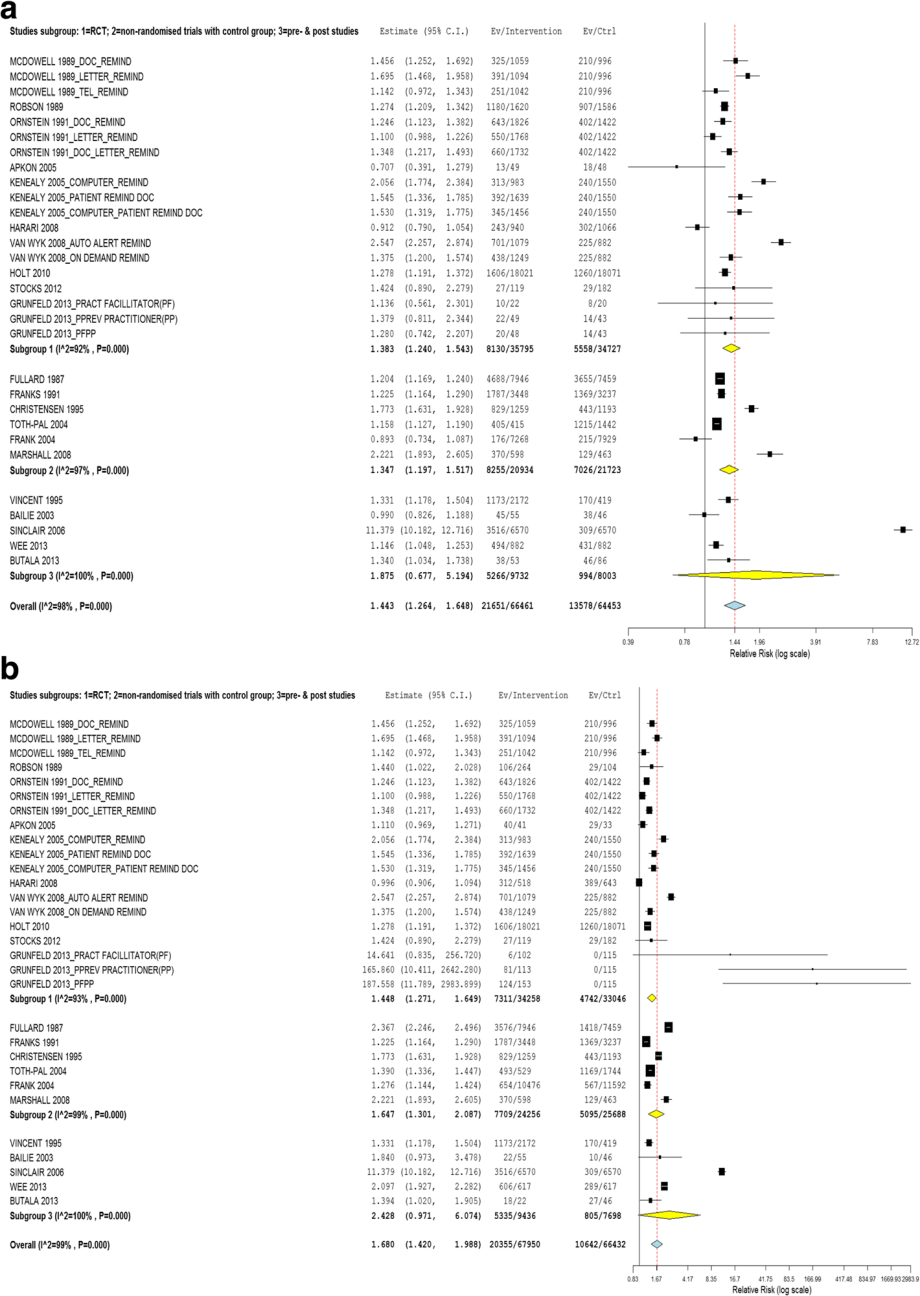
**a** Effect of interventions vs. controls according to study designs (using lowest effect size as outcome measure). **b** Effect of interventions vs. controls according to study designs (using highest effect size as outcome measure)

The quality of studies differed with different study designs. Therefore, we pooled data according to study designs i.e. RCT, non-randomized with controlled group and pre- and post-studies (refer to Fig. 4a & b).

Of the ten studies in the randomized/cluster randomized controlled trials, four studies had three arms of interventions [27, 37, 47, 48], one study had two arms of interventions [49] and others had one arm of intervention. The majority of the studies showed positive effects from the interventions. Two studies, Grunfeld et al. and Apkon et al., reported more than one effect size, and the effects varied from negative to positive effect when pessimistic and optimistic analyses were performed respectively [27, 51]. However, the overall pooled estimate using both pessimistic and optimistic analyses were positive with a RR of 1.383 (95% CI 1.240 to 1.543) and 1.448 (95% 1.271 to 1.649), respectively (refer to Fig. 4a & b, subgroup 1).

All of the non-randomized trials with controlled groups and the pre- and post-studies were significantly in favour of the intervention, except for one study each from both groups when the pessimistic analysis was performed [55, 56]. One study from the pre- and post-study remained ineffective even when an optimistic analysis was performed [56]. The pooled estimate of the effect for non-randomized trials with controlled groups in both pessimistic (RR 1.347; 95% CI 1.197 to 1.517) and optimistic analyses (RR 1.647; 95% CI 1.301 to 2.087) were significantly in favour of the interventions. For the pre- and post-studies the pooled estimate of the effect was not significant in both pessimistic (RR 1.875; 95% CI 0.677 to 5.194) and optimistic analyses (RR 2.428; 95% CI 0.971 to 6.074) (refer to Fig. 4a & b, subgroup 2 and 3).

There was significant heterogeneity between the studies in all three groups of study designs. The I^2^ for all were more than 90%.

#### Effects of types of interventions

**Fig. 5:**
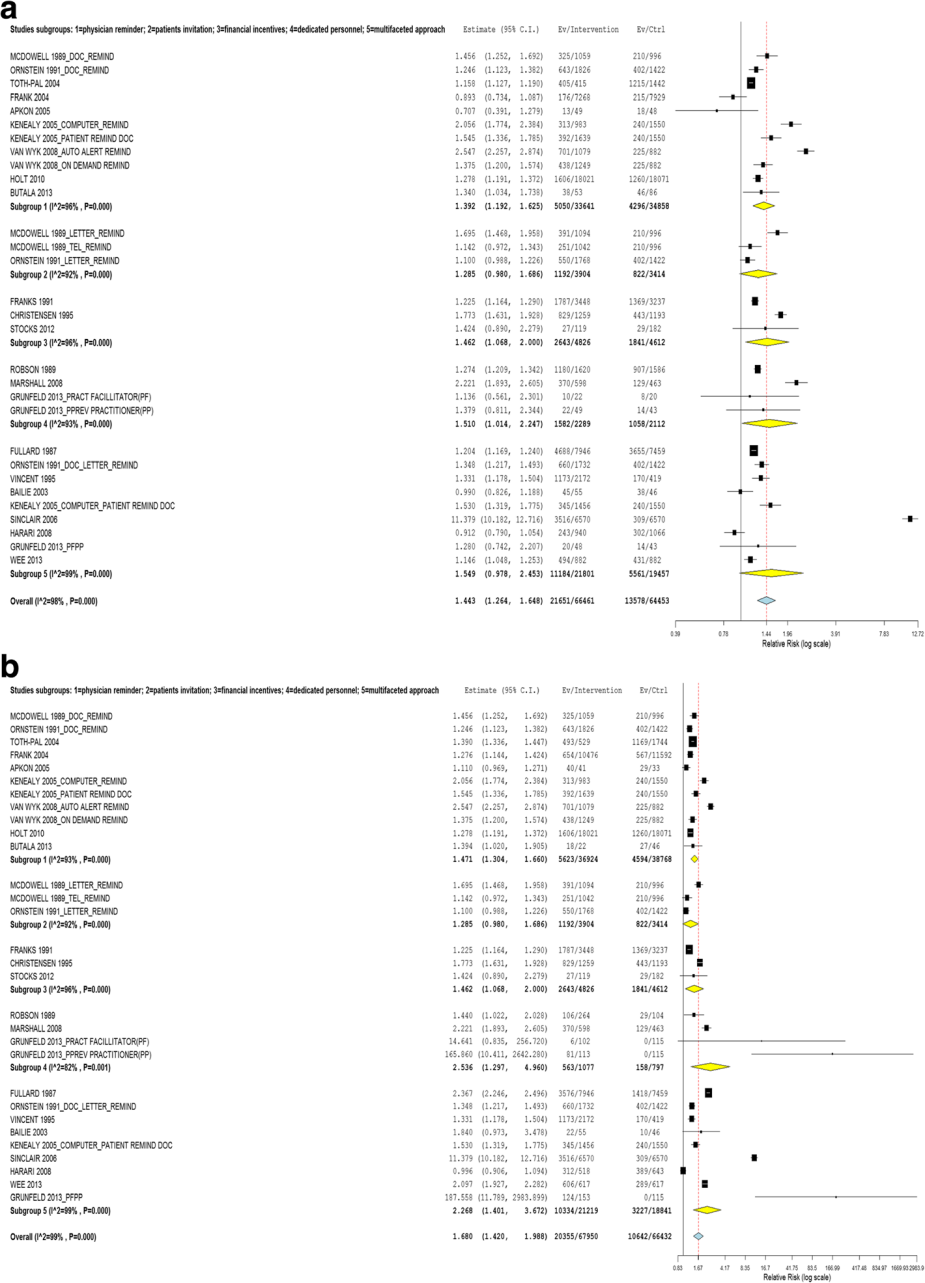
**a** Effect of types of interventions vs. controls (using lowest effect size as outcome measure). **b** Effect of types of interventions vs. controls (using highest effect size as outcome measure)

Using physician reminders (RR 1.392; 95% CI 1.192 to 1.625 in pessimistic analysis and RR 1.471; 95% CI 1.304, 1.660 in optimistic analysis), providing financial incentives (RR 1.462; 95% CI 1.068 to 2.000) and using dedicated personnel (RR 1.510; 95% CI 1.014 to 2.247 in pessimistic analysis and 2.536; 95% CI 1.297 to 4.960 in optimistic analysis) for screening significantly increased the uptake of CVD risk factors screening, compared to the controlled groups. Interventions using multifaceted approaches were effective when optimistic analysis was performed (RR 2.268; 95% CI 1.401, 3.672) but not when pessimistic analysis was performed (RR 1.549; 95% CI 0.978, 2.453). Patient invitations were not effective in increasing the uptake of CVD risk factors screening (RR1.285; 95% CI 0.980, 1.686) (refer to Fig. 5a & b).

In the intervention using physician reminders, six studies used computer-based screen alert system reminders [47, 49, 51, 54, 55, 60], and two studies used paper-based reminders [48, 57]. One study used two approaches, one arm used computer-based screen alert system reminders while in the other arm, physicians were reminded when patients handed the completed diabetes risk self-assessment forms to them [37]. All studies were significantly in favour of the intervention [37, 47–49, 54, 57, 60] except for two [51, 55]. A study by Frank et al. showed a significant positive effect in the optimistic analysis but not in the pessimistic analysis [55]. A study by Apkon et al. showed effects were not significant in both optimistic and pessimistic analyses [51]. (refer to Fig. 5a & b, Subgroup 1).

In the interventions using financial incentives for screening, all studies [59, 62] were significantly in favour of the intervention except for the study by Stocks et al. [58]. Stocks et al. used shopping vouchers as rewards for screening while the other two studies offered free or subsidized screening as the intervention (refer to Fig. 5a & b, Subgroup 3).

All three studies that used dedicated personnel as the intervention to increase screening uptake were conducted at clinics [27, 50, 61]. Two studies used dedicated personnel (project nurse and health promotion nurse) to deliver the screening [50, 61]. Their tasks were to invite, follow-up and deliver the preventive care service. One study used two different approaches; one used dedicated personnel to deliver the screening while the other used a practice facilitator to help the organization improve the system and implement changes for better care [27]. The first two studies that used dedicated personnel to deliver screening showed positive effects in both optimistic and pessimistic analysis [50, 61]. The third study that used dedicated personnel to deliver screening was effective in the optimistic analysis; however, using dedicated personnel targeted at the organizational level was not [27] (refer to Fig. 5a & b, Subgroup 4).

The multifaceted approach was shown to be effective in increasing the screening uptake in the optimistic analysis (RR 2.268; 95% CI 1.401 to 3.672) but not in the pessimistic analysis (RR 1.549; 95% CI 0.978 to 2.453) (refer to Fig. 5a & b, Subgroup 5). There were only two studies that used patient invitations to increase uptake of CVD risk factors screening. The trend was towards a positive effect but it was not significant (RR 1.285; 95% CI 0.980 to 1.686) (refer to Fig. 5a & b, Subgroup 2).

All 21 studies were conducted in primary care settings except for one study from Singapore that was conducted in a housing estate. We have included this community study in the analysis as the objective of this review was to determine the effectiveness of intervention strategies carried out in primary care settings or the community. Removal of this study from the analysis did not alter the effect size nor the statistical significance, although there were magnitude changes in the estimates and CI (refer to Additional file 4 for comparison of the effect size by including and excluding this study in the analysis).

Meta-regression analysis showed that the effect size of CVD risk factors screening uptake was not associated with study designs, types of population and types of interventions in both optimistic and pessimistic analyses.

## Discussion

### Summary of principal findings

We set out to determine the effectiveness of interventions aimed to increase uptake of CVD risk factors screening. Overall, our results showed that the interventions were effective in increasing CVD risk factors screening uptake. This effect size of the uptake was not associated with the study design, types of population nor types of interventions. Effective interventions that increased screening participation included: using physician reminders, use of dedicated personnel to deliver screening and providing financial incentives for screening. Multifaceted approaches were effective when optimistic analysis was performed.

### Interpretation of the findings and comparison with previous findings

We obtained 21 articles for analysis of which 9 were the results from the initial search and 16 were from the forward and backward bibliography and citation checks of the search. The cross checking of bibliographies and citations led to increased yield. This was especially true when an article was cited in a review paper; by going through the studies included in the review paper, we found more papers relevant to our study. Thus, the back and forth citation search could be a better way of tracking relevant articles.

Our results showed that studies with lower quality (pre- and post-studies) had larger effect size (RR ranged from 1.875 to 2.428) but lower precision compared with studies with higher quality such as the non-randomized trials with controlled groups (RR ranged from 1.347 to 1.647) and randomized controlled trials (RR ranged from 1.383 to 1.448). This is expected as studies with better methodology had lower effect size but higher precision, which was consistent with the literature and suggested quality assessment of papers was useful [66].

The heterogeneity observed was significant and high for all the meta-analyses performed. This was expected given the diverse population, settings, study designs, interventions and risk factors measured [66, 67]. Despite this, the results for study effects were robust in one direction (refer to Figs. 3a & b, 4a & b, and 5a & b). This implied that the results could possibly be generalized to various populations [66].

The meta-regression was performed to explore whether the differences in study designs, type of populations and types of intervention could explain the heterogeneity seen in the meta-analysis. Meta-regression is used to explore associations between study-level features and the outcome. For example, the quality of study design can result in artefactual variation. There may also be true differences in effects arising from associations with differences in study population (for instance variation in disease severity) or intervention. Such effect modification may help identify participants for whom the intervention is likely to produce benefit [46]. In this meta-regression analysis, it was found that study designs, types of population and types of intervention did not influence the effect size.

The risk factors targeted for screening were heterogenous and ranged from single to multiple CVD risk factors. There were also interventions that involved other preventive services such as vaccination. By performing two sets of meta-analysis on the highest and lowest uptake rate for each intervention, a range of effect size was provided to clearly demonstrate the effectiveness of interventions across various risk factors.

We found using physician reminders, dedicated personnel or financial incentives for screening were effective interventions. Our study extends the evidence from the previous systematic review by Jepson et al., where the types of interventions previously found to be effective in cancer screening seem to have similar effects in CVD risk factors screening [28]. This suggests that people’s health behaviour towards interventions to improve screening was similar regardless of the condition they had.

Although using physician reminders can increase CVD risk factors screening uptake rate, its effect is limited to patients attending the clinic for other reasons. For interventions using financial incentives to improve screening uptake rates, our result was consistent with the results of Jepson’s review [28]. Both reviews showed that interventions using reduced cost or free screening increased uptake but not those providing incentives such as shopping vouchers, gifts or transportation incentives. The effect of free or subsidized screening is likely to be different depending on the way in which health services are funded. When free screening is provided by existing health-care systems, added rewards do not provide a further effect on the uptake of screening [58].

Dedicated personnel can be used to deliver the screening or to facilitate screening uptake at organizational level. This review found that using dedicated personnel to deliver the screening was effective in increasing CVD risk factors screening uptake; the effect was uncertain for using dedicated personnel targeted at the organizational level as there was only one study researching this intervention. The use of dedicated personnel (non-physician providers) to increase preventive activities has been shown to be effective in previous literature for adult immunization and cancer screening [68, 69]. This intervention requires system resources and support such as organisational change in staffing and clinical procedures. Although using dedicated personnel at organizational level such as practice facilitator has been shown to be effective in improving the use of evidence-based guideline in primary care and preventive care performance [70, 71], the intervention did not consistently show positive results in other studies [27, 72]. This highlights the challenge in the implementation of this intervention which may vary from practice to practice.

For intervention using a multifaceted approach, our results shown inconsistent results in the effectiveness of this intervention. The intervention was effective in optimistic analysis but not when a pessimistic model was used. A systematic review by Jepson et al. reported some evidence in the effectiveness of multiple interventions aimed at individuals or physicians and interventions aimed at both physicians and individuals in increasing screening uptake [28]. Further studies are needed to confirm the effectiveness of multifaceted approaches in increasing the uptake of CVD risk factors screening.

In contrast with other reviews for breast, cervical and colorectal cancer screening [73–75], an invitation to patients either by telephone or letter did not show a significant effect in increasing CVD risk factors screening uptake, although a positive trend was observed. Due to the small samples (two studies and three comparisons), it is difficult to conclude the effectiveness of this intervention for CVD risks factor screening. With the global use of information technology with mobile phones, telephone invitation could be used as a mode for such invitations. There was an increased use of mobile text messages that have been shown to be effective in delivering reminders for adherence to treatment and appointments in health-care services [76]. Thus, mobile text messages might be useful as a mode of invitation for screening. However, we did not find any study using this mode as an intervention for CVD risk factors screening.

### Implications for policy and practice

Our results show that active recruitment targeted at any level, either individual, health-care professional, or health-care system were effective in increasing CVD risk factors screening uptake. However, the intervention one chooses would depend on the practice resources and support and the target of coverage within a set time frame. For example, physician reminders would not be applicable to individuals who did not attend the clinic, and they could potentially be the most at risk group. Provision of free screening can be effective in health-care systems where participants have to pay for screening. Using dedicated personnel to deliver screening was effective, but the cost and human resources demand would be high.

### Strengths and weaknesses of this review

Our review has included both randomized and non-randomized controlled trials as well as pre-and post-studies interventions to provide more comprehensive views of various interventions aimed to increase the uptake of CVD risk factors screening. We believe that each of these studies can contribute useful information to the review. Although the non-randomized and non-controlled trials could inflate the effect size of the interventions, the meta-regression we performed did not show any significant association between study designs and the effect size of the CVD risk factors screening uptake.

There are several limitations in this review. At study level, there was a high risk of bias for blinding of participants and personnel in all the studies. However, this was unavoidable due to the nature of the interventions. At review level, we have employed an extensive search strategy. However, we limited it to publications in the English language, due to limited resources. Hence, the analysis should be treated with caution. In addition, we did not identify any unpublished trials; thus publication bias could not be examined. Other interventions such as providing incentives to health practices might be useful, but we could not find any of such studies.

We did not include cost-effectiveness of interventions in this review which is an important area to look into when choosing an intervention. This will be a useful area to explore in future reviews.

## Conclusion

Physician reminders and providing financial incentives were effective in influencing the provider’s and patient’s behaviour to increase CVD risk factors screening uptake. At organizational level, using dedicated personnel to deliver the screening was found to be effective.

## Additional files

Additional file 1: Characteristics of included study. (XLSX 36 kb)
Additional file 2: Judgement of risk of bias and risk of bias summary of individual studies. (XLSX 42 kb)
Additional file 3: The TIDieR checklist. (XLSX 63 kb)
Additional file 4: Comparison of the effect size by including and excluding the community study. (DOCX 12 kb)

## Abbreviations

95% CI: 95% confidence interval
BMI: Body mass index
BP: Blood pressure
CVD: Cardiovascular diseases
MeSH: Medical subject headings
RCTs: Randomized controlled trials
RR: Relative risk
TIDieR: Template for Intervention Description and Replication
WC: Waist circumference
