# Supplementary material for: Are interventions to increase the uptake of screening for cardiovascular disease risk factors effective? A systematic review and meta-analysis
Source: BMC Fam Pract. 2017 Jan 17;18:4. doi: 10.1186/s12875-016-0579-8 (PMC5240221; doi:10.1186/s12875-016-0579-8)
Supplement: Additional file 4: — Comparison of the effect size by including and excluding the community study. (DOCX 12 kb) [file 12875_2016_579_MOESM4_ESM.docx]

**Additional File 4 Comparison of the effect size by including or excluding the community study (by Wee et al)**

|  | Lowest effect size as outcome  (pessimistic analysis) | | Highest effect size as outcome  (optimistic analysis) | |
| --- | --- | --- | --- | --- |
|  | Effeect size for all the 21 studies  Relative risk  (95%CI) | Effect size after excluding the study by Wee et al.  Relative risk  (95%CI) | Effeect size for all the 21 studies  Relative risk  (95%CI) | Effect size after excluding the study by Wee et al.  Relative risk  (95%CI) |
| Effect of interventions vs. control group | 1.443  (1.264, 1.648) | 1.455  (1.269, 1.669) | 1.680  (1.420, 1.988) | 1.667  (1.401, 1.984) |
| Effect of interventions vs. control group by study design (subgroup 3 Pre & post study) | 1.875  (0.677, 5.194) | 2.121  (0.568, 7.920) | 2.428  (0.971, 6.074) | 2.510  (0.623, 10.122) |
| Effect of types of interventions vs. control group (Subgroup 5 multifaceted approach) | 1.549  (0.970, 2.453) | 1.608  (0.928, 2.789) | 2.268  (1.401, 3.672) | 2.350  (1.318, 4.190) |
